# Fluorescence-guided ureter mapping in colorectal surgery: a systematic review of intraureteral ICG and emerging renal fluorophores

**DOI:** 10.3389/fsurg.2026.1734869

**Published:** 2026-03-10

**Authors:** Alexis Litchinko, Quentin Denost, Barbara Noiret, Frederic Ris, Michel Adamina

**Affiliations:** 1 Division of Digestive Surgery, University Hospitals of Fribourg, Fribourg, Switzerland; 2 Division of Digestive Surgery, University Hospitals of Geneva, Fribourg, Switzerland; 3 Department of Surgery, Bordeaux Colorectal Academy, Bordeaux, France; 4 Department of Medicine, University of Fribourg, Fribourg, Switzerland; 5 Department of Medical and Surgical Specialties, Faculty of Science and Medicine, University of Fribourg, Fribourg, Switzerland; 6 Faculty of Medicine, University of Basel, Basel, Switzerland

**Keywords:** colorectal surgery, CW800-CA, fluorescence imaging, indocyanine green (ICG), minimally invasive surgery, near-infrared (NIR) fluorophores, ureter mapping, ureteral injury

## Abstract

**Background:**

Ureteral injury is a severe complication in colorectal surgery, with an incidence up to 2.5%. Despite meticulous surgical technique, intraoperative ureter identification can be challenging, particularly in cases involving prior operations or extensive inflammation. Indocyanine green (ICG) fluorescence imaging has emerged as a promising adjunct to enhance ureter visualization. In parallel, novel fluorophores with renal excretion properties (e.g., CW800-CA, ZW800-1) are undergoing investigation to avoid routine ureteral catheterization. This systematic review evaluates the efficacy, safety, and clinical impact of ICG-based and emerging fluorescence approaches for ureter identification in colorectal surgery.

**Methods:**

A systematic literature search was performed in PubMed and Embase, up to March 2025, following PRISMA 2020 guidelines. Studies assessing intraoperative ureter identification via ICG fluorescence or other near-infrared fluorophores during adult colorectal surgery were included. Exclusion criteria comprised pediatric populations, non-colorectal procedures, reviews, editorials, and animal experiments. Primary outcomes were ureter visualization rate and intraoperative ureteral injury rate, while secondary outcomes included procedure-related complications, operative time, adverse effects, and preliminary cost data.

**Results:**

Ten studies comprising 716 patients undergoing colorectal surgery with ICG fluorescence imaging were analyzed. Ureter visualization rates ranged from 95.3% to 100%, with most studies reporting a rate of 100%. No ICG-related complications were documented. ICG administration was primarily via cystoscopy with intra-ureteral injection or ureteral catheterization, predominantly in laparoscopic and robot-assisted procedures. Mean duration of the cystoscopy varied from 7 to 29 min. Improved intraoperative ureter identification compared with conventional visualization was reported in available comparative studies.

**Conclusion:**

ICG fluorescence imaging safely and effectively enhances intraoperative ureter visualization during colorectal surgery, potentially reducing the risk of ureteral injuries. However, the need for routine ureteral catheterization prolongs procedure duration. Emerging renally excreted fluorophores may eliminate the need for catheterization and should be prioritized in future clinical trials.

**Systematic Review Registration:**

PROSPERO [CRD420250653992].

## Graphical Abstract

**Figure:**
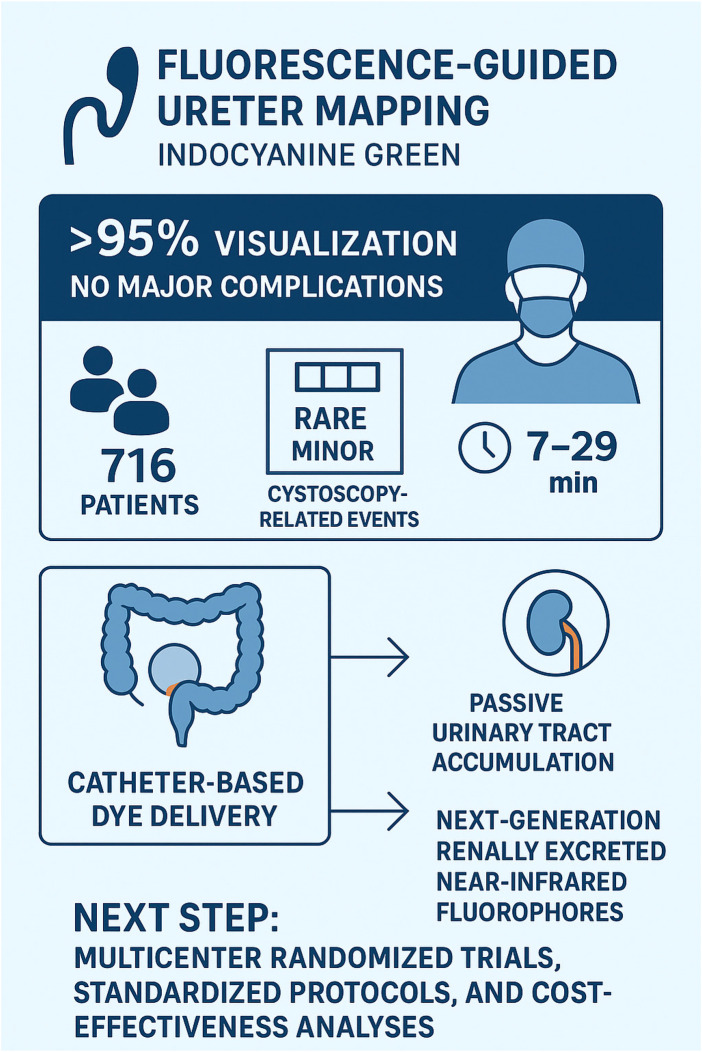

## 1 Introduction

Ureteral injury is a rare but serious complication of colorectal surgery, particularly during pelvic resections, with an incidence reaching up to 2.5% (1–3). These injuries are often missed intraoperatively and can lead to significant postoperative morbidity, including renal dysfunction, ureteral strictures, reinterventions, and, in severe cases, nephrectomy (4). Accurate intraoperative identification of the ureters is therefore essential to prevent iatrogenic damage, especially in anatomically complex cases such as inflammatory bowel disease, extensive adhesions, or locally advanced malignancies.

Traditional strategies, such as preoperative ureteral stenting or intraoperative palpation, have been widely used to aid in ureter identification. While effective in open procedures, these methods are limited in minimally invasive surgery, where tactile feedback is reduced. Routine ureteral catheterization also adds procedural time and carries its own risks, including mucosal trauma, urinary tract infections, and transient hematuria (5, 6). Despite these precautions, ureteral injuries may still occur, underscoring the need for more reliable, real-time visualization tools.

Fluorescence-guided imaging has emerged as a novel adjunct to improve intraoperative navigation. Indocyanine green (ICG) fluorescence, in particular, has demonstrated clinical utility across multiple surgical disciplines, including hepatobiliary, vascular, and colorectal surgery. When administered intraureterally, ICG binds to the urothelium and enables real-time visualization of the ureters under near-infrared fluorescence (NIRF) imaging systems (7, 8). This technique offers a potential alternative to prophylactic stenting by enhancing anatomical delineation without the drawbacks associated with prolonged ureteral catheterization.

Despite increasing clinical adoption, the efficacy and utility of ICG fluorescence for ureter identification in colorectal surgery remain subjects of debate. The available evidence is largely derived from small, heterogeneous studies, often with variations in administration technique, imaging platforms, and outcome measures. Additionally, concerns have been raised regarding the procedural complexity of intraureteral dye instillation and its impact on operative workflow (1, 7). Intravenous ICG delivery has been proposed as a simpler method, though its renal clearance is minimal, limiting its effectiveness for direct ureter visualization. Meanwhile, newer renally excreted fluorophores such as CW800-CA and ZW800-1 are being explored in preclinical models and early clinical trials, offering the promise of ureter mapping without the need for catheterization (9–11).

This systematic review aims to synthesize current clinical evidence regarding ICG fluorescence imaging for intraoperative ureteral identification in colorectal surgery. By evaluating efficacy, safety, and procedural considerations, this review seeks to determine whether fluorescence guidance constitutes a viable alternative to traditional techniques and to highlight directions for future research and technological innovation.

## 2 Materials and methods

### 2.1 Study design and registration

This systematic review was conducted in accordance with the Preferred Reporting Items for Systematic Reviews and Meta-Analyses (PRISMA) 2020 guidelines to ensure methodological rigor and transparency. The study protocol was registered in PROSPERO (Registration ID: CRD420250653992) before the initiation of the literature search (12).

### 2.2 Search strategy

A systematic literature search was conducted in PubMed and Embase, library to identify studies evaluating the use of indocyanine green (ICG) fluorescence imaging for intraoperative ureter identification in colorectal surgery. The search strategy incorporated a combination of Medical Subject Headings (MeSH), Emtree terms, and free-text keywords related to ICG fluorescence, ureter identification, and colorectal surgery. Boolean operators were used to optimize sensitivity and specificity. The search was restricted to studies published between 2015 and February 2025, with language filters applied to include only articles in English and French. Additional relevant studies were identified by manually screening the reference lists of included articles.

### 2.3 Eligibility criteria

Studies were selected according to predefined inclusion and exclusion criteria based on the Population, Intervention, Comparison, Outcome, and Study Design (PICOS) framework (13). Eligible studies included those involving adult (>18 yo) patients undergoing colorectal surgery in which ICG fluorescence was used for intraoperative ureter visualization and iatrogenic prevention. Primary outcomes assessed were the efficacy of ICG in ureteral identification and the incidence of intraoperative ureteral injuries. Secondary outcomes included operative time, procedure-related complications, adverse events, and exploratory preliminary cost considerations related to ureteral catheterization and operative workflow, when reported. Studies focusing on pediatric populations, non-colorectal procedures, animal models, or experimental techniques not yet applied in clinical practice were excluded. Review articles, editorials, conference abstracts without full-text availability, and opinion pieces were also excluded.

### 2.4 Study selection and data extraction

Two independent reviewers screened and selected the studies according to the predefined eligibility criteria (A.L. & B.N.). Titles and abstracts were first reviewed to exclude irrelevant studies, followed by a full-text assessment of potentially eligible articles. Disagreements between reviewers were resolved by discussion or, if necessary, by consulting a third reviewer. Data extraction was performed using a standardized collection form, including study characteristics, patient demographics, surgical indications, ICG administration protocols, fluorescence detection techniques, and reported outcomes.

### 2.5 Quality assessment and data synthesis

The quality of included studies was assessed using the ROBINS-I tool for non-randomized studies (14). The level of evidence was evaluated using the Grading of Recommendations, Assessment, Development, and Evaluations (GRADE) approach (15). Due to the heterogeneity of study designs, patient populations, and ICG administration protocols, a narrative synthesis was performed instead of a meta-analysis. This approach allows for a qualitative assessment of trends and clinical implications across different studies.

### 2.6 Ethical considerations

This study is a secondary analysis of previously published data and did not require institutional review board approval. No external funding was received, and no conflicts of interest were reported by the authors.

## 3 Results

### 3.1 Study selection

The systematic search yielded 318 studies after duplicate removal. Following title and abstract screening, 45 full-text articles were assessed for eligibility. Among them, 10 studies met the inclusion criteria and were included in the final qualitative synthesis. The PRISMA flow diagram (Table 1) illustrates the study selection process.

**Table 1:** PRISMA FLowchat.

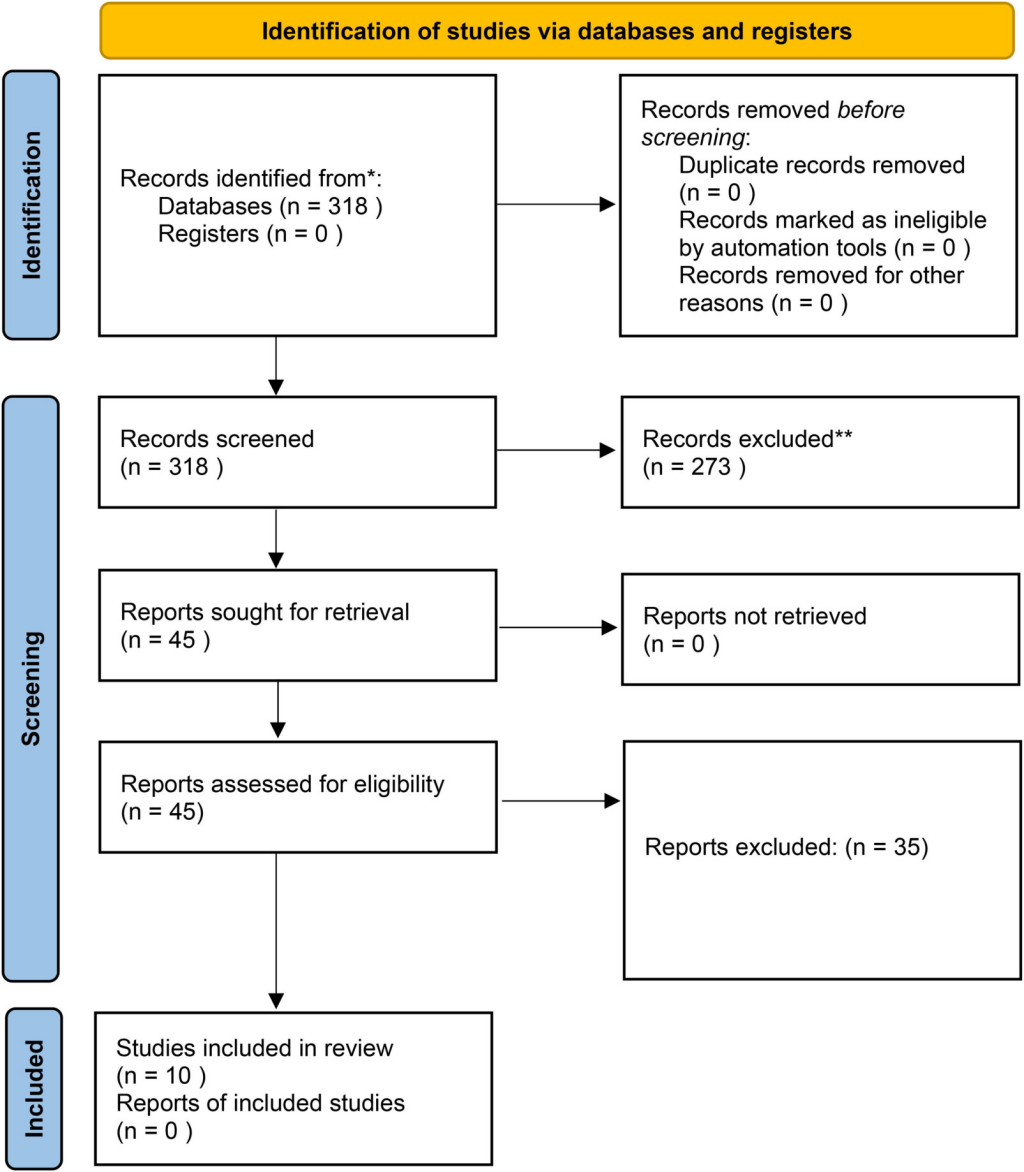

### 3.2 Study characteristics

The 10 included studies were published between 2019 and 2024. Study populations ranged from 2 to 312 patients. Indocyanine green (ICG) was administered intraureterally in most studies, primarily via cystoscopic retrograde injection or direct intraureteral instillation. Three studies used Near-Infrared Fluorescent Ureteral Catheters (NIRFUC), which rely on catheter-based fluorescence rather than dye instillation. Most studies reported bilateral intraureteral ICG administration to ensure symmetrical visualization, particularly in complex pelvic dissections. Ureteral stents were systematically used in 60% of the studies, selectively used in 10%, and not used in 30%. Most procedures were minimally invasive with laparoscopic (58%), followed by robot-assisted colorectal surgery (42%).

### 3.3 Primary outcomes

#### 3.3.1 Ureteral visualization rate

Fluorescence imaging was predominantly performed using near-infrared platforms. The ureteral visualization success rate with ICG fluorescence was consistently high across studies, ranging from 95.3% to 100%. Overall, 80% of the included studies reported complete visualization of both ureters. Indocyanine green was predominantly administered via intraureteral injection, in association with high visualization rates. Comparative effectiveness based on surgical approaches (laparoscopic vs. robot-assisted) was not clearly quantified; however, robotic procedures benefited from enhanced NIR imaging capabilities provided by technologies such as Firefly®. A detailed summary of study characteristics and ICG administration protocols is presented in Table 2.

**Table 2:** Study characteristics and ICG administration protocols.

Reference	Year	Country	Study type	Number of patients with ICG	ICG administration method	wUreteral stent use
Mandovra et al. (24)	2019	India	Retrospective cohort	30	Intra-ureteral injection of 5 mg ICG diluted in 2 ml of distilled water	No
Ryu et al. (25)	2021	Japan	Retrospective case series	11	Intravenous & intra-ureteral injection of ICG for ureter and vessel navigation	Yes
White et al. (26)	2021	USA	Retrospective case series	16	Direct intra-ureteral injection of 4 mg ICG in 4 ml NaCl	No
Ryu et al. (27)	2021	Japan	Pilot study	20	Near-Infrared Fluorescent Ureteral Catheter (NIRFUC)	Yes
Soriano et al. (28)	2022	USA	Prospective cohort	83	Intra-ureteral injection of 5 ml ICG (2.5 mg/ml)	20 with stent 63 without stent
Satish et al. (29)	2022	India	Case reports	2	Cystoscopic retrograde injection of 2.5 mg ICG per ureter	Yes
Ryu et al. (16)	2022	Japan	Cohort comparative study	122	Near-Infrared Fluorescent Ureteral Catheter (NIRFUC)	Yes
Rodriguez-Zentner et al. (30)	2023	Panama	Case series	30	Intra-ureteral injection of ICG	Yes
Ryu et al. (31)	2024	Japan	Retrospective study	141	Near-Infrared Fluorescent Ureteral Catheter (NIRFUC)	Yes
Rogers et al. (8)	2024	USA	Retrospective comparative cohort	312	Injection of 5 ml ICG solution (2.5 mg/ml) per ureter	Yes

#### 3.3.2 Ureteral injury rate

The incidence of intraoperative ureteral injury was low across studies using ICG fluorescence, although comparative data were limited. In studies reporting comparative outcomes, lower ureteral injury rates were observed with ICG fluorescence compared with conventional visualization in high-risk surgical settings (3.2% vs. 7.8%, *p* < 0.05). No ICG-related adverse events were reported. Lower ureteral injury rates were primarily reported in cohorts including patients with prior pelvic surgery, advanced malignancy, or inflammatory bowel disease.

### 3.4 Secondary outcomes

#### 3.4.1 Procedure-related complications & operative time

The most frequently reported complications were related to cystoscopy itself. Indeed, transient hematuria, occurring in 5.4% of patients receiving intraureteral ICG administration. Other minor complications included dysuria (2.5%) and urinary tract infections (1.8%). No adverse events related to ICG administration were reported across the studies. Intraureteral administration of ICG via cystoscopic catheterization increased operative time by an average of 7–29 min across studies. No studies evaluating intravenous ICG administration were included. Thus, no comparison of operative time or visualization rates between intraureteral and intravenous ICG routes or simple ureteral stent could be performed. None of the included studies specifically investigated the use of renally excreted fluorophores (CW800-CA, ZW800-1, or ICG derivatives). Therefore, no conclusions can currently be drawn regarding their efficacy or safety in colorectal surgery. Further studies are required to evaluate the potential of these fluorophores for intraoperative ureter visualization without catheterization (Table 3).

**Table 3:** Indications and surgical procedures.

Reference	Year	Surgical Indications (included/studied)	Surgical Procedures	Vizualisation Success rate	Mean Cystoscopic Procedure Duration (minutes)	ICG-Related Complications
Mandovra et al. (24)	2019	Various colorectal indications (30/30)	Laparoscopic colorectal procedures (30)	100%	7	None
Ryu et al. (25)	2021	Various colorectal indications (11/14)	Laparoscopic colorectal procedures (11)	100%	14	None
White et al. (26)	2021	Various colorectal indications (16/16)	Laparoscopic colorectal surgery (16)	94%	11.5	None
Ryu et al. (27)	2021	Various colorectal indications (20/20)	Laparoscopic colorectal surgery (20)	100%	Unknow	None
Soriano et al. (28)	2022	Diverticulitis (43), Colorectal cancer (30), Other (10)	Robot-assisted left colectomy (45), Sigmoidectomy (38)	97% right ureter 100% left ureter	4 without stent place-Ment 13.5 with stent placement	None
Satish et al. (29)	2022	Rectal cancer (2)	Low anterior resection (1), Hartmann's procedure (1)	100%	Unknowm	None
Ryu et al. (16)	2022	T4 colorectal cancers (122/122)	Laparoscopic colorectal resection (122)	100%	Unknown	None
Rodriguez-Zentner et al. (30)	2023	Colorectal cancer cases (30/30)	Laparoscopic colorectal procedures (30)	100%	22.4	None
Ryu et al. (31)	2024	Colorectal cancer and colorectal cancer recurrence (141/141)	Laparoscopic colorectal procedures (141)	100%	12	None
Rogers et al. (8)	2024	Colorectal cancer and Diverticulitis (556/312)	Laparoscopic colectomy (100), Robot-assisted (456)	95.3%	29	None

### 3.5 Quality assessment and risk of bias

**Figure 1:**
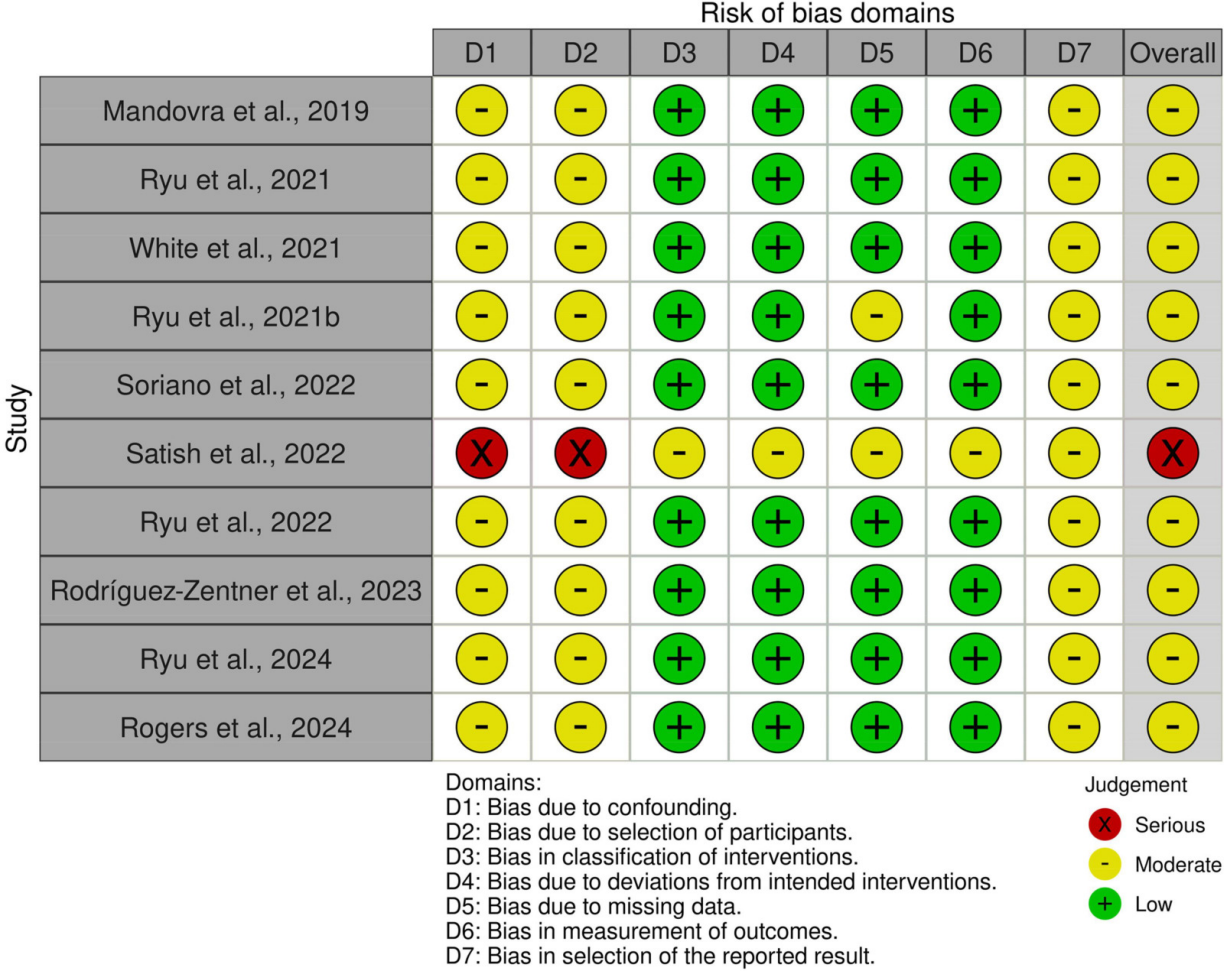
Quality assessment and risk of bias (ROBINS-I).

The risk of bias for the included studies was assessed using the ROBINS-I tool for observational studies. Among the 10 studies evaluated, 9 were observational studies (retrospective or prospective cohorts and case series), and 1 was a pilot study (16). Most of these studies (7 out of 10) were classified as having a moderate risk of bias, primarily due to heterogeneity in the fluorescence imaging protocols, variability in patient selection criteria, and the absence of comparator groups. No randomized controlled trials were included in this review. According to the GRADE approach, the level of evidence supporting the efficacy of ICG fluorescence for ureter identification in colorectal surgery was moderate. Evidence regarding cost-effectiveness was insufficient and remains very limited. All included studies were observational, and none were randomized; most had a moderate risk of bias based on ROBINS-I assessment (Figure 1).

## 4 Discussion

### 4.1 Clinical benefits of ICG fluorescence

Ureteral injury, though rare in colorectal surgery, carries significant morbidity, particularly in complex pelvic dissections. Indocyanine green (ICG) fluorescence provides real-time, high-contrast ureter visualization, especially valuable in minimally invasive procedures where tactile feedback is absent. Across 10 studies (716 patients), intraureteral ICG achieved visualization rates of 95.3%–100% (pooled mean 98.4%), with 80% of studies reporting complete bilateral delineation. Comparative data suggest lower ureteral injury rates with ICG (3.2% vs. 7.8%, *p* < 0.05) in high-risk cases, although derived from observational comparative data. Protocol heterogeneity—dose, timing, stenting strategy, and imaging platform—limits comparability, but safety was consistently high, with no ICG-specific toxicity and only minor cystoscopy-related events (e.g., transient hematuria, 5.4%). The main drawback is the additional 7–29 min required for catheter placement.

### 4.2 Limitations and barriers

Catheter-based intraureteral instillation remains the standard delivery method, prolonging surgery and requiring cystoscopic access. Protocol variation (ICG 0.25–5 mg, pre- vs. intraoperative) and differences in surgical approach and imaging systems complicate pooled interpretation. All studies were observational with moderate risk of bias, as no randomized controlled trials exist (5). While robotic platforms (e.g., Firefly®) may enhance imaging, they are not universally available, particularly in lower-resource settings. Also, routine bilateral catheterization may expose the contralateral ureter, sometimes not directly involved in the dissection, to unnecessary manipulation, with a theoretical risk of ureteral injury or urinary complications. This aspect should be addressed in future protocols.

### 4.3 Future directions and emerging technologies

Renally excreted near-infrared (NIR) fluorophores constitute a major technological advancement, designed to enable real-time ureteral mapping following intravenous administration, thereby overcoming the logistical limitations of catheter-based delivery. Unlike ICG, which is hepatically cleared, agents such as CW800-CA, ZW800-1, and IRDye 800BK are eliminated via the kidneys, allowing intravenous administration and passive urinary tract accumulation (9–11, 17–19).

ZW800-1: A zwitterionic dye with minimal non-specific binding, providing a high signal-to-background ratio. Detectable in ureters within 10 min of injection, with signal persisting up to 60 min. CW800-CA: PEGylated cyanine dye with excellent renal clearance and stable fluorescence; shows greater signal stability but slower onset than ZW800-1.

In terms of kinetic, ZW800-1 peaks faster but fades sooner, whereas CW800-CA provides prolonged stability—properties that may guide selection based on operative duration. These agents could eliminate cystoscopy, streamline workflow, and reduce complications. However, they remain investigational, with no large-scale RCTs, incomplete dosing standardization, and pending regulatory approval. Importantly, dual-agent fluorescence—for example, ICG for bowel perfusion plus ZW800-1 for ureter mapping—offers the possibility of simultaneous vascular and urinary tract visualization without procedural delays (18, 20).

### 4.4 Economic considerations

Future cost-effectiveness analyses integrating both direct expenditures and potential savings from injury prevention will be essential to support reimbursement and guideline integration (21–23). Contemporary ICG-based protocols frequently rely on cystoscopy-guided ureteral catheterization, entailing direct procedural expenditures ranging from approximately 300–800 CHF per case. Beyond these immediate costs, such approaches necessitate additional anesthesia and urology team involvement, thereby increasing operative time and resource utilization. Moreover, potential downstream expenses may arise from cystoscopy-related adverse events, further challenging the overall cost-effectiveness of the technique. The 7–29 min procedural delay may reduce operating room throughput, particularly in high-volume centers. IV-administered renally excreted fluorophores could remove these bottlenecks, but their cost-effectiveness remains unproven without formal economic modeling. Importantly, cost data were heterogeneous, indirect, and not derived from formal economic evaluations, precluding definitive cost-effectiveness conclusions.

Future studies should evaluate direct expenditures (dye, equipment, OR time) alongside indirect savings from prevented injuries, shorter hospital stays, and avoidance of litigation. This evidence will be essential to inform reimbursement, purchasing decisions, and guideline integration.

## 5 Conclusion

ICG fluorescence represents a safe and effective adjunct for intraoperative ureteral identification, achieving visualization rates above 95% across studies with negligible dye-related complications. Its main limitations—catheter dependence, protocol heterogeneity, and lack of randomized evidence—currently restrict widespread adoption. Emerging intravenously administered, renally excreted NIR fluorophores hold the potential to simplify workflow and expand access to fluorescence-guided surgery. Multicenter randomized trials integrating clinical, economic, and workflow outcomes are warranted to define the optimal strategy for intraoperative ureter mapping in colorectal surgery.

## Data Availability

The original contributions presented in the study are included in the article/Supplementary Material, further inquiries can be directed to the corresponding author.
